# Antimicrobial-Resistance Profile of *Helicobacter pylori*, Obtained from Endoscopic Patients in Bahir Dar, North West Ethiopia

**DOI:** 10.1155/2023/7326288

**Published:** 2023-11-13

**Authors:** Mulat Erkihun, Desalegn Nigatu Chanie, Yesuf Adem Siraj

**Affiliations:** ^1^Department of Medical Laboratory Sciences, School of Health Sciences, College of Medicine and Health Sciences, Debre Tabor University, P.O. Box 272, Debre Tabor, Ethiopia; ^2^Department of Internal Medicine, School of Medicine, College of Medicine and Health Sciences, Bahir Dar University, P.O. Box 79, Bahir Dar, Ethiopia; ^3^University of Campania Luigi Vanvitelli, Caserta, Italy

## Abstract

**Background:**

Antimicrobial resistance for *Helicobacter pylori* infection is a highly emerging problem throughout the world to treat gastric-associated diseases. People living in developing countries are more likely to acquire a *Helicobacter pylori* infection and less likely to gate treatment after infection due to poverty. Therefore, the current study was aimed at determining the magnitude and antibiotic-resistance profile of *Helicobacter pylori* isolated from patients who underwent endoscopic examination.

**Methods:**

A cross-sectional study was conducted from January to May 2019 at endoscopy service-providing health facilities that are found in Bahir Dar, Ethiopia (Gamby teaching general hospital, Kidane Mihret specialty higher clinic, and Eyasta specialty higher clinic). Data were collected using a pretested, structured questionnaire. Antibiotic susceptibility of *Helicobacter pylori* isolates from gastric biopsies was determined. Data were analyzed using SPSS version 23.

**Results:**

The 17.8% proportion of *Helicobacter pylori* was isolated from 135 endoscopy-examined patients (24/135). The majority of isolates (71% of 17/24) were from males, while only 29% of 7/24) were from females. Antimicrobial-resistance of *Helicobacter pylori* was high to all commonly prescribed antibiotics: amoxicillin and metronidazole (91.7%), clarithromycin and ciprofloxacin (66.7% each), and tetracycline (37.5%).

**Conclusion:**

*Helicobacter pylori* isolates from the current study participants were rather low in number. But the highest antibiotic-resistance profile of *Helicobacter pylori* was observed. Therefore, these findings alarmingly indicate that routine antimicrobial susceptibility testing against *Helicobacter pylori* isolates is crucial for better patient management.

## 1. Introduction

The *Helicobacter pylori* (*H. pylori*) is a Gram-negative, four-to-seven polar flagellated bacterium with spiral or curved morphology [1]. It has a microaerophilic oxygen requirement. The bacterium was first isolated from the gastric culture in 1982 by Australian scientists Robin Warren and Barry Marshall [2]. It is classified under the kingdom Monera, domain Bacteria, phylum Proteobacteria, class Epsilonproteobacteria, order *Campylobacterales*, family *Helicobactericeae,* and genus *Helicobacter* [3].

*H. pylori* are the causative agent for gastric-associated diseases like gastric cancer and mucosa-associated lymphoid tissue (MALT) lymphoma [4–6]. Based on its global impact, the International Agency for Cancer Research and the World Health Organization (WHO) grouped *H. pylori* as a class I biological carcinogenic agent [7].

It is possible to be cured of the *H. pylori* infection with a proper treatment regimen. Among the treatment regimens, combinations containing clarithromycin and/or amoxicillin, metronidazole, tetracycline, and ciprofloxacin are the most commonly utilized antibiotics with proton pump inhibitors [6, 8, 9]. But its antimicrobial resistance is worsening the treatment outcome [10, 11].

Based on clarithromycin resistance, *H. pylori* was categorized as one of the World Health Organization (WHO) priority pathogens listed for research and development of new antibiotics [12].

Although global amoxicillin resistance is low, there are *H. pylori* strains with high levels of amoxicillin resistance that are related with beta-lactamase synthesis. Penicillin-binding protien1A point mutation has been associated to low-level amoxicillin resistance [13].

The emergence of multidrug-resistant (MDR) strains of *H. pylori* is a global health concern.

Studies across the world are approving that *H. pylori* is becoming among the superbugs and recommending antimicrobial sensitivity testing prior to eradication therapy [14].

Particularly in low- and middle-income countries, the problem is worsening due to a lack of treatment options that include bismuth salt, including regimens, diagnostic tools, and skills for case detection [14–16].

Because of the worldwide increase in antibiotic resistance, most guidelines have recommended antimicrobial susceptibility testing to guide *H. pylori* eradication treatment [17].

However, due to the fastidious nature of *H. pylori* and the lack of a simple and cost-effective testing method, antimicrobial susceptibility testing for *H. pylori* is not available in Ethiopia. Profiling regional or population-specific antibiogram patterns is critical in directing the creation of effective empiric therapy regimens in such circumstances. As a result, the purpose of this study is to present the resistance profile of *H. pylori* in the study area.

## 2. Materials and Methods

### 2.1. Study Area

This study was carried out at health facilities in Bahir Dar City that offered endoscopic services. The Gamby teaching general hospital, Kidane Mihret specialty higher clinic, and Eyasta specialty higher clinic were among these institutions. Bahir Dar is 565 km from Addis Ababa in the country's northwest. It is 1784 meters above sea level and has latitude 11.6, longitude 37.3833 11.5742°N, and longitude 37.3614°E coordinates.

### 2.2. Study Design and Period

An institutional-based cross-sectional study was undertaken on patients with gastric-associated disorders who had endoscopic examinations from January to May of 2019.

### 2.3. Sociodemographic and Clinical Data Collection

During interviews, structured questioning was utilized to collect sociodemographic data from research participants. Participants' clinical state was also examined during endoscopic examinations by checking their patient charts and discussing with their physicians. Participants who were receiving eradication or antiacid therapy were barred from participation in the study.

### 2.4. Specimen Collection, Transport, and Processing

The lowest compliance for the Sydney protocol was obtained during biopsy specimen collection, according to the Sydney protocol. During an endoscopic examination of different anatomical areas of the stomach, two to four gastric biopsy specimens were taken from suspected gastric-associated diseased patients using sterile endoscope forceps [18]. During specimen collection, any potential risk of an adverse outcome was assessed, and a clinical decision was made. To improve patient comfort and endoscopic performance, sedation for endoscopic procedures was maintained by delivering 1 ml (5 mg) of diazepam via intravenous root and 4-5 ml of lidocaine spray via rinsing the mouth. Biopsy specimens were instantly inoculated into brain-heart infusion broth and immediately delivered to Bahir Dar University's College of Medical and Health Sciences' Medical Microbiology Laboratory. After 48 hours of incubation, inoculated broths were subcultured onto Charcoal Cefoperazone Deoxycholate Agar (CCDA) and Campy Agar supplemented with 5% sheep blood [19]. Secondary subculture was performed after 10 days of incubation for negative broths. In candle jars with a microaerobic environment (5% O_2_, 10% CO_2_, and 85% N_2_), inoculated broth bottles and culture plates were incubated. Gas-generating kits and anaerobic catalysts (OXOID, England) were used to keep the environment microaerobic. Subcultures were checked for bacterial development until the third day of inoculation. Furthermore, negative culture results were only issued after inspection of cultures every 24 hours for ten days of incubation. The antimicrobial sensitivity test was the standardized Kirby–Bauer technique (disc diffusion) test, which adhered to McNulty et al. recommendation. On Mueller–Hinton agar bases with 10% blood, a suspension equivalent to the McFarland standard was used. Then, commonly used antibiotics were added, namely, metronidazole (MTZ 5 *μ*g), amoxicillin (AMX 10 *μ*g), tetracycline (TET 30 *μ*g), erythromycin (ERY 15 *μ*g), clarithromycin (CLT 5 *μ*g), ciprofloxacin (CPR 5 *μ*g), and amoxicillin with clavulanic or Augmentin (AUG 20/10 *μ*g).

### 2.5. Bacterial Identification

The selective media that allow the growth of *H. pylori* species were used. A range of identification assays were used on preliminary bacterial colonies, including Gram staining, oxidase, catalase, and urease tests. Being microaerophilic and the need for selective and enriched media are features of *H. pylori* [20].

### 2.6. Quality Control

The data gathering questionnaire was designed to be simple to use. Each participant's format was reviewed for completeness during the data collecting period. Standard operating procedures (SOPs) were also followed to ensure the quality of the research from preanalytical to postanalytical. In each sterilization operation, *Geobacillus stearothermophilus* (ATCC 7953) was autoclaved and incubated for growth detection. For the sterility test, 5% of each batch of media preparation was incubated overnight at 35–37°C. The *Campylobacter jejuni* (ATCC 33560) strain, which exhibits growth characteristics similar to those of *H. pylori* strains, was used to evaluate the culture media and incubation environment.

### 2.7. Data Processing and Analysis

The collected data were validated for correctness before being entered into Epi Data software version 3.1. The data were then imported into and analyzed with SPSS version 23. Data analysis was illustrated using frequency tables, and its relationship to relevant factors was examined. *P* values less than 0.05 were considered statistically significant.

## 3. Results

### 3.1. Sociodemographic Characteristic of Study Subjects

During the study period, 135 gastric-associated patients who underwent endoscopic examinations were included. There were 59.3% (80/135) males and 40.7% (55/135) females among the study participants. The participants' average age was 45 years, with a range of 18 to 88 years. The majority of study participants (81.5%) had low incomes, whereas those with medium and high incomes were 17% and 1.5%, respectively.

### 3.2. *H. pylori* Isolates from Gastroantral Biopsies

In this investigation, the prevalence of *H. pylori* isolated from gastric-associated patient biopsies was 17.8% (24/135). Positive study participants were 71% (17/24) males and 29% (7/24) females, with a significant proportion of positivity in the 30–60 age range (54.2% (13/24). Rural individuals were infected with *H. pylori* at a greater rate (75% of the total of 24), while urban dwellers had only 25% (6 of the total of 24). The majority of individuals in the culture-positive trial (75%, 18/24) were married. Furthermore, all of the culture-positive results (24/24) came from study participants with low income.

### 3.3. Antimicrobial-Resistance Profile of *H. pylori* Isolates

All 24 isolates of *H. pylori* were tested for commonly used antimicrobials. The antimicrobial susceptibility profile of *H. pylori* isolates showed resistance to more than one antimicrobial agent. For instance, antimicrobial-resistant *H. pylori* isolates were detected against amoxicillin and metronidazole (91.7% each), clarithromycin, ciprofloxacin, and erythromycin (66.7% each), while tetracycline was only at 37.5%. Resistance to commonly prescribed antibiotics was found to be more prevalent in patients with any type of gastritis, including gastric ulcers and atrophied gastritis. *H. pylori* isolates from atrophied gastritis patients showed resistance to amoxicillin (33.3%), metronidazole, clarithromycin (29.2% each), ciprofloxacin (20.8%), and tetracycline (12.5%) (Table 1).

The proportion of multidrug-resistant (MDR) *H. pylori* isolates in the current study was 54.2% (13/24), 45% (11/24), 29% (7/24), and 13% (3/24) for combinations of 3, 4, 5, and 6 commonly prescribed antibiotics, respectively (Table 2).

## 4. Discussion

Antimicrobial resistance is currently a major global problem [21, 22]. As antibiotic resistance reduces treatment efficacy, it is time to consider routine susceptibility testing to guide individual patient treatment and monitor antibiotic resistance. The burden of resistant strains of *H. pylori* remains a major challenge in the course of the treatment regimen [23–25]. Triple or quadruple therapy is frequently prescribed by most clinicians. This strategy consists of two antibiotics plus a proton pump inhibitor or/and bismuth salt [26]. The current study also found that antimicrobial resistance is becoming more difficult in the study area.

In the current study, all *H. pylori* isolates from patients with gastric-associated diseases were resistant to more than one antibiotic. Majorities of *H. pylori* isolates were resistant to amoxicillin (91.7%), although global *H. pylori* resistance to amoxicillin is extremely contradictory and low [27, 28]. This might be an indication for evidence of the irrational use of amoxicillin for the treatment of different gastrointestinal infectious diseases.

In this study, the resistance rates of commonly prescribed antibiotics such as amoxicillin and metronidazole were 91.7% each, clarithromycin and ciprofloxacin were 66.7% each, and tetracycline was 37.5%. Similarly, a study conducted in the western part of Nigeria indicated that *H. pylori* isolates were resistant to common antibiotics: amoxicillin (66%), erythromycin (78%), metronidazole (95%), and tetracycline (100%) [29].

The clarithromycin resistance finding in this study indicates that the treatment regimen in the study area should be in concordance with the regimen protocol suggested for clarithromycin resistance >20% regions. Furthermore, WHO is alarmingly aware that *H. pylori* with clarithromycin resistance are the priority pathogens for new treatment protocol development [12].

However, a previous study conducted in the United States revealed that a lower proportion of *H. pylori* isolates (25.1%), clarithromycin (12.9%), and amoxicillin (0.9%) were resistant. In contrast to our findings, the same study reported a very high susceptibility to amoxicillin [23]. Furthermore, different studies reported a lower resistance proportion to amoxicillin [30]. Studies indicated that high-level amoxicillin resistance is associated with beta-lactamase production in *H. pylori*, and low-level amoxicillin resistance is linked to a point mutation on penicillin-binding protien1A [13]. It seems that there is a high prevalence of beta-lactamase producing *H. pylori* strains at the study area. The broad use of this antibiotic to treat different infectious diseases could contribute to the development of more resistant strains.

In contrast to our study findings, a study conducted on adult dyspeptic patients at Tikur Anbassa University Hospital, Ethiopia, indicated that all *H. pylori* isolates were sensitive to clarithromycin, tetracycline, and erythromycin, and only 6% of the isolates were resistant to amoxicillin, which was a much lower resistance rate than our study finding [31].

In the present study, all strains of *H. pylori* isolates were multidrug resistant. Among all, thirteen (54.2%) are resistance for three drugs and three of all showed a MDR profile for six commonly prescribed antibiotics. From the study that was conducted to determine the frequency of MDR *H. pylori* strains in Iran, primary, double, and multidrug resistance frequencies were assessed. Among the resistant strains: the rates of double and multiple drug resistance phenotypes were 22.6% (19/84) and 34.5% (29/84), respectively, which reviled lesser MDR prevalence compared with this study [32].

## 5. Limitations

This study considered only severely ill gastric-associated diseased participants who were sent for endoscopic examinations and might have a repeated eradication treatment history. But gastric complaints or patients in outpatient departments were being seen at growing rates which did not included in this study. Another limitation was the inability to get previously done culture-based studies on *H. pylori* infection antimicrobial resistance profiles at the study area for better comparison.

## 6. Conclusions

Antimicrobial resistance of *H. pylori* isolates is so alarming that susceptibility testing before the initiation of treatment is vital for effective treatment of *H. pylori*-associated gastroduodenal diseases. Adherence to the World Health Organization's (WHO) recommended standard treatment regimens in regions with a history of high clarithromycin resistance will warrant better treatment outcomes. Further studies should be carried out on resistance mechanisms for better management of *H. pylori*-induced gastric-associated diseases. Further molecular investigations for the clarithromycin-resistance mechanism shall be conducted. Quinolones containing treatment regimens has to be also supported by antimicrobial sensitivity testing for better eradication treatment outcomes.

## Figures and Tables

**Table 1:** Antimicrobial-resistant profile of *H. pylori* isolates from patients attending endoscopic examination.

*H. pylori*isolates from endoscopy findings	Susceptibility status	AMX	MTZ	CLM	TET	CPR	ERY	AUG
		*N*(%)	*N*(%)	*N*(%)	*N*(%)	*N*(%)	*N*(%)	*N*(%)
Normal mucosa	Sensitive	0	0	0	2 (8.3)	0	0	0
	Intermediate	0	0	0	0	0	0	0
	Resistance	4 (16.7)	4 (16.7)	4 (16.7)	2 (8.3)	4 (16.7)	4 (16.7)	4 (16.7)

Atrophic pan gastritis	Sensitive	1 (4.2)	2 (8.3)	2 (8.3)	5 (20.8)	2 (8.3)	1 (4.2)	1 (4.2)
	Intermediate	0	0	0	1 (4.2)	2 (8.3)	0	0
	Resistance	8 (33.3)	7 (29.2)	7 (29.2)	3 (12.5)	5 (20.8)	8 (33.3)	8 (33.3)

Non atrophic pan gastritis	Sensitive	0	0	0	1 (4.2)	1 (4.2)	0	0
	Intermediate	0	0	0	0	0	0	1 (4.2)
	Resistance	1 (4.2)	1 (4.2)	1 (4.2)	0	0	1 (4.2)	0

Antral gastritis	Sensitive	1 (4.2)	0	1 (4.2)	3 (12.5)	2 (8.3)	0	1 (4.2)
	Intermediate	0	0	0	0	0	0	1 (4.2)
	Resistance	3 (12.5)	3 (12.5)	2 (8.3)	0	1 (4.2)	3 (12.5)	1 (4.2)

Any gastritis and gastric ulcer	Sensitive	0	0	3 (12.5)	2 (8.3)	1 (4.2)	1 (4.2)	1 (4.2)
	Intermediate	0	0	2 (8.3)	1 (4.2)	0	0	0
	Resistance	7 (29.2)	7 (29.2)	2 (8.3)	4 (16.7)	6 (25)	6 (25)	6 (25)

Total resistance		22 (91.7)	22 (91.7)	16 (66.7)	9 (37.5)	16 (66.7)	22 (91.7)	19 (79.2)

**Table 2:** Multidrug-resistance profile of *H. pylori* isolates from patients with gastric-associated diseases attending endoscopic examination.

Antibiotics	*R*_3_ * N* (%)	*R*_4_ * N* (%)	*R*_5_ * N* (%)	*R*_6_ * N* (%)
CLM + AMX + MTZ	13 (54.2)			
CLM + AMX + CPR	10 (41.7)			
CLM + AMX + AUG	12 (50.0)			
CLM + AMX + TET	6 (23.0)			
CLM + MTZ + CPR	10 (41.7)			
CLM + MTZ + TET	6 (23.0)			
CLM + MTZ + AUG	12 (50.0)			
CLM + CPR + TET	4 (17.0)			
CLM + CPR + AUG	8 (33.0)			
CLM + TET + AUG	5 (21.0)			
CLM + AMX + MTZ + TET		5 (21.0)		
CLM + AMX + MTZ + CPR		9 (37.0)		
CLM + AMX + MTZ + AUG		11 (45.8)		
CLM + AMX + TET + CPR		4 (17.0)		
CLM + AMX + CPR + AUG		8 (33.3)		
CLM + AMX + MTZ + CPR + TET			3 (13.0)	
CLM + AMX + MTZ + CPR + AUG			7 (29.2)	
CLM + AMX + TET + CPR + AUG			3 (13.0)	
CLM + AMX + MTZ + CPR + TET + AUG				3 (13.0)

*R *_3_, *R*_4_, *R*_5_, and *R*_6_ are resistant for 3, 4, 5, and 6 antimicrobials.

## Data Availability

The study includes a summary of patient information, linked factors with *H. pylori* association, and antibiotic susceptibility profiles in the form of texts and tables. Authors can provide raw data upon reasonable request.
